# Assessment of serum tenascin-C and growth differentiation factor-15 among type 2 diabetes mellitus patients with and without acute coronary syndrome

**DOI:** 10.5937/jomb0-24662

**Published:** 2020-10-02

**Authors:** Murugaiyan Vasanthi, Prashant Shankarrao Adole, Vinay Ramakrishna Pandit, Kolar Vishwanath Vinod

**Affiliations:** 1 Jawaharlal Institute of Postgraduate Medical Education and Research (JIPMER), Department of Biochemistry, Pondicherry, India; 2 Jawaharlal Institute of Postgraduate Medical Education and Research (JIPMER), Department of Emergency Medicine and Trauma, Pondicherry, India; 3 Jawaharlal Institute of Postgraduate Medical Education and Research (JIPMER), Department of Medicine, Pondicherry, India

**Keywords:** type 2 diabetes mellitus, acute coronary syndrome, tenascin-C, growth differentiation factor-15, faktor diferencijacije rasta-15, tenascin-C, akutni koronarni sindrom, dijabetes tip 2

## Abstract

**Background:**

High prevalence of type 2 diabetes mellitus (T2DM) is associated with a higher prevalence of acute coronary syndrome (ACS). Inflammation is one of the important contributors to the pathogenesis and complications of coronary atherosclerotic plaque. Growth Differentiation Factor-15 (GDF-15) and Tenascin-C (TNC) play an important role in the initiation of atherosclerotic plaque as well as its rupture. The aim of the study was to evaluate the association between serum GDF-15, TNC, and the risk of ACS among T2DM patients.

**Methods:**

Anthropometric parameters, routine biochemical investigations like liver and renal function tests, lipid profile, and Creatine Kinase-Total (CK-T), Creatine Kinase-MB (CK-MB) were measured in 42 T2DM patients with ACS and 42 T2DM patients. Serum GDF-15 and TNC were measured by Human Sandwich-ELISA kits.

**Results:**

Serum GDF-15 and TNC levels were significantly higher in T2DM patients with ACS as compared to T2DM patients. Serum GDF-15 was significantly correlated with waist circumference, diastolic blood pressure, pulse, serum CK-T, and CK-MB. Serum TNC was significantly correlated with the pulse, serum CK-T, CK-MB, high-density lipoprotein-cholesterol, and blood urea nitro GEN. Multivariate linear regression analysis showed that waist circumference was independently positively associated with serum GDF-15.

**Conclusions:**

T2DM patients with higher serum GDF-15 and TNC levels were at higher risk of acute coronary syndrome independent of other cardiovascular risk factors.

## Abstract (sr)

**Uvod:**

Visoka prevalenca dijabetes melitusa tipa 2 (T2DM) povezana je sa većom prevalencom akutnog koronarnog sindroma (ACS). Upala je jedna od važnih stavki u patogenezi i komplikacijama koronarnog aterosklerotskog plaka. Faktor diferencijacije rasta-15 (GDF-15) i tenascin-C (TNC) igraju važnu ulogu u pokretanju aterosklerotskog plaka, ali i njegovom pucanju. Cilj studije bio je da se proceni povezanost između seruma GDF-15, TNC-a i rizika od ACS-a kod pacijenata sa T2DM.

**Metode:**

Kod 42 pacijenta koji boluju od T2DM sa ACS i 42 pacijenta sa T2DM izmereni su antropometrijski parametri, rutinska biohemijska ispitivanja poput testova jetre i funkcije, lipidni profil i ukupna kreatin kinaza (CK-T), kreatin kinaza-MB (CK-MB). Serum GDF-15 i TNC su mereni pomoću humanih sendvič-ELISA kitova.

**Rezultati:**

Serumski nivo GDF-15 i TNC su bili značajno viši kod T2DM pacijenata sa ACS u poređenju sa T2DM pacijentima. Serumski GDF-15 je bio značajno povezan sa obimom struka, dijastolnim krvnim pritiskom, pulsom, serumskim CK-T i CK-MB. TNC u serumu je bio značajno povezan sa pulsom, serumskim CK-T, CK-MB, lipoprotein holesterolom visoke gustine i azotom uree u krvi. Multivarijantna linearna regresiona analiza pokazala je da je obim struka nezavisno pozitivno povezan sa serumskim GDF-15.

**Zaključak:**

Pacijenti sa T2DM sa većem nivoom GDF-15 i TNC u serumu su bili izloženi većem riziku od akutnog koronarnog sindroma nezavisno od ostalih kardiovaskularnih faktora rizika.

## Introduction

Type 2 diabetes mellitus (T2DM) has emerged as a major public health concern all over the world and most importantly, India has been recognized as the Capital of diabetes by World Health Organi zation (WHO) [1]. As T2DM shares several risk factors in common with coronary artery disease, an increase in the frequency of diabetes concomitantly increases the incidence of coronary artery disease. Diabetics patients are at increased risk for developing coronary artery disease than non-diabetic counterparts of similar age, gender, and ethnicity [2]. Cardiovascular disease reports for nearly 80% of premature deaths in diabetic individuals. Since inflammation plays a crucial role in the manifestation and evolution of diabetes and diabetes-related cardiovascular complications, assessment of inflammatory biomarkers may help in the diagnosis of future cardiovascular risk among T2DM patients [3].

Tenascin-C (TNC) is a glycoprotein of an extracellular matrix. Normally, TNC is not present in myocardium except at the chorda tendineae of papillary muscles, but it is found to be expressed under pathological conditions. It is involved in multiple functions such as apoptosis, cell proliferation, differentiation, and migration. In patients with the acute coronary syndrome (ACS), TNC increases the expression and activity of matrix metalloproteinases which trigger degradation of collagen and weaken the attachment of cardiomyocytes from connective tissue ultimately resulting in atherosclerotic plaque instability [4]. TNC expression was found to rise with the increasing plaque instability resulting in its rupture [5]. Many studies have shown that TNC has been accepted as a predictor of left ventricular remodelling and prognostic marker of ACS [6] [7] [8].

Growth Differentiation Factor-15 (GDF-15) comes under the transforming growth factor cytokine family. Normally, GDF-15 is weakly expressed in cardiac tissue and the high level was observed only in pathological conditions such as tissue injury and inflammation. GDF-15 has a role in cellular functions, organs renovation, differentiation, inflammation, and apoptosis [3] [9]. Elevated GDF-15 protects endothelial cells from high glucose-mediated cellular injury by initiating phosphoinositide 3kinase/Protein kinase B/endothelial nitric oxide synthase pathway and attenuating Nuclear Factor-kB/c-Jun N-terminal kinases activation [10]. Though GDF-15 is not a cardiac-specific protein and elevated in many other tissues, namely liver, kidney, and lung, increased levels of GDF-15 have been observed in the setting of myocardial infarction, heart failure and cardiomyopathy. GDF-15 is also an independent predictor of insulin resistance and abnormal glucose homeostasis in obese individuals, thereby it may provide a link between obesity, diabetes and cardiovascular risk [11] [12].

In T2DM patients, elevated levels of TNC may predispose them for plaque rupture and, ultimately, ACS. Increased serum GDF-15 level in T2DM patients with ACS neutralizes the deleterious effect of inflammation. The limited data on serum TNC and GDF-15 levels among T2DM with ACS is available in the literature. Therefore, the objectives of the study were to assess and compare serum TNC and GDF-15 levels and find its association with personal and family history, biochemical investigations among T2DM with and without ACS and to determine whether serum TNC and GDF-15 can be used to predict the risk of ACS among T2DM patients.

## Materials and Methods

### Study participants

The study was conducted in the Jawaharlal Institute of Postgraduate Medical Education and Research, (JIPMER) Pondicherry from 2017-2019. Approval of the Institute Research Council and the Institute Human Ethics Committee was taken (JIP/IEC/2017/0338 Dated 11/11/2017). All procedures were in accordance with ethical standards of the responsible committee on human experimentation (Institutional and national) and with the Helsinki Declaration of 1964, as revised in 2013. Informed consent was obtained from all participants included in the study. T2DM patients with (ACS) including Unstable Angina (UA), Non-ST elevation myocardial infarction (NSTEMI) and ST elevation myocardial infarction (STEMI) were recruited as cases in the study. Age, gender and duration of diabetes matched T2DM patients without ACS were recruited as controls. ACS is defined as the presence of the following: 1) clinical presentation, 2) electrocardiographic changes, and 3) cardiac enzyme elevation [13]. Patients with the chronic renal disease with serum creatinine level > 221 μmol/L, liver cirrhosis, congestive heart failure, chronic lung diseases, symptomatic peripheral vascular diseases, neoplasm and chronic infections were excluded.

### Clinical and Biochemical Parameters

Personal and medical histories for all subjects were recorded. Height, weight, waist circumference, and seated blood pressure were measured by the same observer. Body mass index (BMI) was calculated as weight (kg) divided by the square of height (meter). Under the strict aseptic condition, fasting five-millilitre venous blood was collected from all subjects enrolled in the study and used for routine biochemistry investigations and TNC and GDF-15 analysis. Routine biochemistry investigations i.e., cho le sterol, triglycerides, High-Density Lipoprotein-Cho lesterol (HDL-C), Low-Density Lipoprotein - Cholesterol (LDL-C), Creatine Kinase-Total (CK-T), Creatine Kinase-MB (CK-MB), blood urea nitrogen (BUN), creatinine were assessed by a fully automated analyzer by an appropriate method. Serum levels of TNC and GDF-15 were estimated by using Human Sandwich-ELISA kits (Elab Science) according to the manufacturer's instructions. The intra-assay and inter-assay CV for serum TNC was 4.65 and 5.45 respectively. The intra-assay and inter-assay CV serum GDF-15 was 5.47 and 5.59, respectively.

### Statistical analysis

Normality of data distribution was assessed by the Kolmogorov-Smirnov test. The continuous data such as age, weight, BMI, Waist circumference, cholesterol, triglycerides, HDL-C, LDL-C, CK-T, CK-MB, BUN, creatinine, TNC, and GDF-15 levels were expressed as mean ± standard deviation (SD). The distribution of categorical data such as smoking habits and alcoholics were expressed as percentages. Student's t-test or Wilcoxon ranks-sum test was used to look for a significant difference in variables between the two groups. Pearson or Spearman's correlation coefficient was used to find the correlation among serum TNC, GDF-15, and biochemical variables. Multivariate linear regression analysis was done to look for the independent association between TNC and GDF-15 with ACS adjusted with parameters that had shown significant correlations with serum TNC and GDF-15 concentration. The value of *P* < 0.05 was considered significant. Statistical analysis and data management were conducted using IBM SPSS Statistics 16 (IBM Corporation, Somers, NY).

## Results

### Baseline clinical characteristics and biochemical measurements

Demographic, clinical, and biochemical data for the cases and controls were shown in Table 1. Among the cases, 71% of patients had ST-segment elevation myocardial infarction (STEMI), 19% of patients had unstable angina (UA), 9% patients had Non-ST-elevation myocardial infarction (NSTEMI). Inferior wall, anterior wall and posterior wall myocardial infarction were found among 10, 6 and one patient, respectively. T2DM patients with ACS had significantly higher height, pulse, CK-total, CK-MB, BUN, creatinine, hemo globin as compared to T2DM patients without ACS. T2DM patients with ACS had significantly lower weight, waist circumference, BMI, diastolic blood pressure, triglycerides as compared to T2DM patients without ACS. There was no significant difference in systolic blood pressure, blood glucose, serum lipid profile except triglycerides among cases and controls.

**Table 1:** Clinical and biochemical properties of the study population. *P* < 0.05. Continuous variables were described as mean ± SD; categorical variables were presented as frequencies. HDL-C, high-density lipoprotein cholesterol; LDL-C, low-density lipoprotein cholesterol; CK-total, creatine kinase-total; CK-MB, creatine kinase-MB; TNC, Tenascin-C; GDF- 15, Growth differentiation Factor-15.

Characteristic	Type 2 Diabetes Mellitus with acute coronary syndrome N = 42 (Mean±SD)	Type 2 Diabetes Mellitus N = 42 (Mean ±SD)	P
Age (years)	57.80 ± 10.28	54.54 ± 10.63	0.827
Weight (kg)	61.95 ± 9.12	67.09 ± 19.69	< 0.001
Height (cm)	163.04 ± 7.79	160.42 ± 15.52	< 0.001
Waist circumference (cm)	89.86 ± 7.99	91.13 ± 10.93	0.044
Body mass index (kg/m^2^)	23.35 ± 3.48	24.65 ± 4.85	0.033
Systolic blood pressure (mmHg)	118.11 ± 17.27	119.76 ± 14.05	0.187
Diastolic blood pressure (mmHg)	73.47 ± 13.04	79.52 ± 6.60	< 0.001
Pulse (per min)	92.73 ± 16.25	76.28 ± 6.24	< 0.001
Duration of Diabetes (years)	5.10 ± 2.91	5.30 ± 3.57	0.188
Glucose (mmol/L)	13.36 ± 5.74	16.38 ± 5.90	0.857
CK-Total (IU/L)	574.42 ± 17.55	75.97 ± 39.94	< 0.001
CK-MB (IU/L)	42.90 ± 7.98	2.88 ± 1.34	< 0.001
Cholesterol (mmol/L)	3.98 ± 1.14	4.44 ± 0.97	0.342
Triglycerides (mmol/L)	1.67 ± 0.75	1.89 ± 1.07	0.025
HDL-C (mmol/L)	0.76 ± 0.18	0.87 ± 0.24	0.056
LDL-C (mmol/L)	2.49 ± 0.91	2.78 ± 0.82	0.508
BUN (mmol/L)	10.15 ± 4.63	7.88 ± 2.38	< 0.001
Creatinine (μmol/L)	91.05 ± 16.80	83.10 ± 42.43	< 0.001
Haemoglobin (g/L)	124.80 ± 20.90	119.20 ± 13.40	0.005
GDF-15 (ng/L)	1155.45 ± 321.01	841.63 ± 477.04	0.011
Tenascin-C (ng/mL)	13.06 ± 9.80	6.16 ± 3.58	< 0.001

### Serum tenascin-C concentration in groups

**Figure 1:**
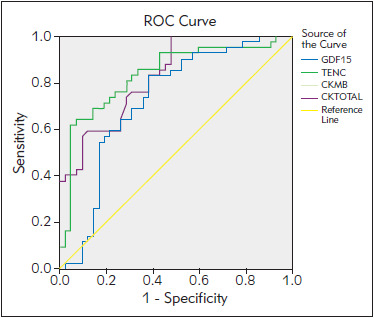
ROC curve for serum TNC (ng/mL) and GDF-15 (pg/mL) discriminatory abilities towards ACS in T2DM patients.

T2DM patients with ACS had significantly higher serum TNC concentration compared to T2DM patients without ACS (13.06 ± 9.80 ng/mL vs 6.16 ± 3.58 ng/mL; P < 0.01). Utilizing ROC curve for the value of TNC and risk of ACS among T2DM patients, it was seen that the greatest increase in risk of ACS was seen at serum TNC concentration more than 6.99 ng/mL (AUC = 0.836, sensitivity of 81% and specificity of 71%, LR= 2.79, P < 0.001) (Figure 1).

### Serum GDF-15 concentration in groups

T2DM patients with ACS had significantly higher serum GDF-15 concentration compared to T2DM patients without ACS (1155.45 ± 321.01 ng/L vs. 841.63 ± 477.04 ng/L, P < 0.01). Utilizing ROC curve for value of GDF-15 and risk of ACS among T2DM patients, it was seen that the greatest increase in risk of ACS was seen at serum GDF-15 concentration more than 1075.78 ng/L (AUC = 0.727, sensitivity of 69%, and specificity of 69%, LR = 2.23, P < 0.001) (Figure 1).

### The correlation between serum TNC, GDF-15 concentration, and clinical parameters

The correlation analysis undertaken on all T2DM patients showed that serum TNC concentration was positively correlated with the pulse (ρ= 0.399, P = 0.001), serum CK-MB (ρ = 0.560, P =0.011), CK-Total (ρ = 0.320, P = 0.021) and BUN (ρ = 0.270, P = 0.013) and negatively correlated with serum HDL-cholesterol (ρ = -0.263, P =0.016). Serum GDF-15 concentration was negatively correlated with diastolic blood pressure (ρ = -0.251, P = 0.022) and positively correlated with pulse (ρ = 0.364, P = 0.001), serum CK-Total (ρ =0.329, P = 0.031), CK-MB (ρ = 0.436, P = 0.014) and waist circumference (ρ = 0.255, P = 0.019) (Table 2). The multivariate linear regression analysis determined that waist circumference was a significant predictor of serum GDF-15 concentration andpulse was significant predictor of serum TNC concentration (Table 3).

**Table 2:** Correlation between tenascin-C, GDF-15, and othervariables. Data are presented as correlation coefficient Rho (ρ)

Parameter	Type 2 Diabetes Mellitus patients with the acute coronary syndrome (n = 42)			
	TNC		GDF-15	
	ρ	P	ρ	P
Waist circumference	-0.063	0.569	0.255	0.019
Diastolic blood pressure	-0.129	0.243	-0.251	0.022
Pulse	0.399	0.001	0.364	0.001
CK-Total	0.320	0.021	0.329	0.031
CK-MB	0.560	0.011	0.436	0.014
HDL-Cholesterol	-0.263	0.016	-0.106	0.336
BUN	0.270	0.013	0.129	0.241

**Table 3:** Predictors of TNC and GDF-15 in the Multivariate linear regression analysis.

Independent Variables	Adjusted Odd Ratio (95% Confidence Interval)	P
GDF-15		
Waist Circumference	4.72 (2.40 – 21.18)	0.014
TNC		
Pulse	0.061 (0.0356 – 0.277)	0.012

## Discussion

The main finding of this study is that serum TNC, GDF-15, and markers of inflammation, are associated with the risk of ACS in patients with T2DM. Further analysis showed a significant positive correlation between serum TNC, GDF-15, CK-Total, and CK-MB in T2DM patients with ACS.

Coronary artery disease is the major cause of morbidity and mortality among diabetic patients. People with diabetes are at greater risk of developing ACS than non-diabetics [14]. An atherosclerotic plaque rupture is a major event responsible for thromboembolic phenomena throughout the body resulting in a stroke, myocardial infarction, etc. Atherosclerotic plaque rupture is a complex process in which inflammation plays an important role. We hypothesized that serum TNC and GDF-15 concentrations in T2DM patients with ACS might reflect the activity of endothelial cells in coronary plaques. Hence, the study aimed to understand the association between serum tenascin-C, GDF-15 levels and risk of the acute coronary syndrome among T2DM patients. The study was conducted on T2DM patients with ACS and age, gender, and duration of diabetes matched T2DM patients without ACS so that there would be no effect of age, gender, and duration of diabetes on study parameters. From the study, we found that there was a significant difference between cases and controls with respect to weight, height, waist circumference, BMI, diastolic blood pressure, pulse, triglycerides, CKtotal, CK-MB, BUN, creatinine and hemo globin. There is no significant difference in duration of diabetes, serum lipid profile (except triglycerides) among cases and controls. Dominguez-Rodriguez et al. [15] found that there was no significant difference in age, gender and standard biochemical results between T2DM patients with and without diabetic cardiomyopathy.

Serum GDF-15 concentration was found to be significantly higher in T2DM patients with ACS as compared to T2DM patients without ACS. Serum GDF-15 was found to be associated with various complications of diabetes. Dominguez-Rodriguez et al. [15] found that GDF-15 levels were a useful and novel tool to screen diabetic cardiomyopathy in asymptomatic patients with T2DM. Li et al. [16] concluded that plasma GDF-15 might serve in early diagnosis, evaluation and prediction of the outcomes of type 2 diabetic nephropathy. Schopfer et al. [17] showed that higher levels of GDF-15 were associated with major cardiovascular events in patients with stable ischemic heart disease. Khan et al. [18] concluded that GDF-15 was a new marker for predicting death and heart failure in Post-AMI patients. Increased GDF-15 was a beneficial response so that it can protect the heart by activating Smad2, Smad3 and ALK4/5/7 receptors. Also, GDF-15 had an antiapoptotic effect against ischemia-reperfusion and reduced the size of myocardial infarction [19].

Serum TNC concentration was found to be significantly higher in T2DM patients with ACS as compared to T2DM patients without ACS. Gaber et al. [20] showed that serum TNC levels were higher in patients with an acute myocardial infarction as compared to healthy volunteers. Kenji et al. [4] showed that TNC may have a role in coronary plaque formation and may be involved in coronary lesions in ACS. Jin-Hu et al. [7] showed that TNC and OX40L act synergistically in coronary plaque formation and may also involve in the pathogenesis of coronary lesion. Sakamoto et al. [5] showed that the TNC level is associated with pathological conditions in ACS, especially the presence of ruptured plaque. TNC expression in the plaque region plays an important role in the initiation of rupture. Increased expression of TNC leads to rupture of plaques, thrombus formation, occlusion of coronary arteries and acute coronary syndrome. Correlation study showed a significant positive correlation of serum TNC and GDF-15 with serum CK-Total and CK-MB. Serum TNC and GDF-15 can be used for the diagnosis of ACS in patients with T2DM as their expression was not found in normal heart and would be expressed more in pathological conditions. Also, the serum level of TNC and GDF-15 was correlated with serum CK-MB which is considered an important marker for the myocardial infarction. ROC analyses showed that serum TNC and GDF-15 had good probability for diagnosis of ACS in T2DM patients with adequate sensitivity and specificity.

This study has limitations. First, diabetic patients were controls that cannot exclude confounding effects such as the effects of diseases itself and drug treatment. Second, the different duration and different treatment of each T2DM patient might incorporate a possible source of selection bias. Third, the study population was relatively small. Finally, the cause-effect relationship between serum GDF-15, TNC levels and ACS was not possible from a crosssectional study.

In conclusion, serum GDF-15 and TNC were significantly elevated in T2DM patients with ACS as compared to T2DM patients without ACS and were positively correlated with serum CK-MB. Hence serum GDF-15 and TNC can be considered as one of the parameters for predicting and diagnosis of an acute coronary event in patients with type 2 diabetes mellitus. However, further research is warranted to confirm by considering large sample size and by conducting longitudinal studies.

*Acknowledgements*. We gratefully acknowledgeall of the patients and Mrs. Durga, a laboratory technician, for her technical support during this study. This work was supported by the JIPMER Intramural Research Committee, Pondicherry, with the fundedgrant (No.: JIP/Res/Intramural/phs2/2017–18, dated01/12/2017). We thank all the examinees for theirparticipation in the current study.

## Conflict of interest statement

The authors stated that they had no conflicts of interest regarding the publication of this article.
